# Athlete’s ECG Made Easy: A Practical Guide to Surviving Everyday Clinical Practice

**DOI:** 10.3390/jcdd11100303

**Published:** 2024-10-01

**Authors:** Valerio Fanale, Andrea Segreti, Chiara Fossati, Giuseppe Di Gioia, Federica Coletti, Simone Pasquale Crispino, Francesco Picarelli, Raffaele Antonelli Incalzi, Rocco Papalia, Fabio Pigozzi, Francesco Grigioni

**Affiliations:** 1 Cardiology Unit, Fondazione Policlinico Universitario Campus Bio-Medico, Via Alvaro del Portillo, 200, 00128 Rome, Italy; 2 Research Unit of Cardiovascular Science, Department of Medicine and Surgery, Università Campus Bio-Medico di Roma, Via Alvaro del Portillo, 21, 00128 Rome, Italy; 3 Department of Movement, Human and Health Sciences, University of Rome “Foro Italico”, Piazza Lauro de Bosis, 15, 00135 Rome, Italy; 4 Institute of Sports Medicine and Science, National Italian Olympic Committee, Largo Piero Gabrielli, 1, 00197 Rome, Italy; 5 Unit of Internal Medicine, Fondazione Policlinico Universitario Campus Bio-Medico, Via Alvaro del Portillo, 200, 00128 Rome, Italy; 6 Research Unit of Orthopaedic and Trauma Surgery, Department of Medicine and Surgery, Università Campus Bio-Medico di Roma, Via Alvaro del Portillo, 21, 00128 Rome, Italy; 7 Department of Orthopaedic and Trauma Surgery, Università Campus Bio-Medico di Roma, Via Alvaro del Portillo, 21, 00128 Rome, Italy

**Keywords:** Athlete, Athlete’s heart, ECG pattern

## Abstract

Electrocardiogram modifications in athletes are common and usually reflect structural and electrical heart adaptations to regular physical training, known as the athlete’s heart. However, these electrical modifications sometimes overlap with electrocardiogram findings that are characteristic of various heart diseases. A missed or incorrect diagnosis can significantly impact a young athlete’s life and potentially have fatal consequences during exercise, such as sudden cardiac death, which is the leading cause of death in athletes. Therefore, it is crucial to correctly distinguish between expected exercise-related electrocardiogram changes in an athlete and several electrocardiogram abnormalities that may indicate underlying heart disease. This review aims to serve as a practical guide for cardiologists and sports clinicians, helping to define normal and physiology-induced electrocardiogram findings from those borderlines or pathological, and indicating when further investigations are necessary. Therefore, the possible athlete’s electrocardiogram findings, including rhythm or myocardial adaptation, will be analyzed here, focusing mainly on the differentiation from pathological findings.

## 1. Introduction

**Figure 1:**
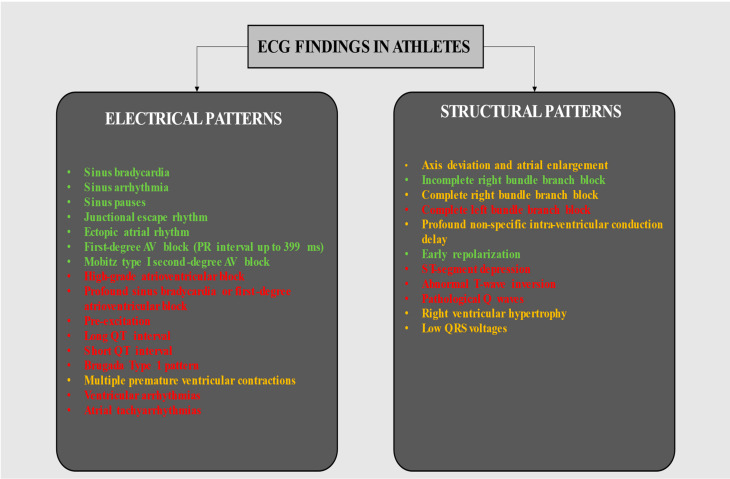
Classification of electrocardiographic findings in athletes based on potential underlying cardiac disease. The figure categorizes ECG findings commonly observed in athletes, distinguishing between those that are typically benign and related to physiological adaptations of the athlete’s heart and those that may suggest underlying pathological conditions requiring further investigation. Green, yellow, and red font colors indicate normal, borderline, and abnormal ECG findings, respectively. Abbreviations: AV, atrioventricular block.

Screening enables the early detection of cardiovascular disease, allowing lifestyle modifications and therapeutic intervention [1]. A preparticipation screening inclusive of history, physical examination, baseline 12-lead electrocardiogram (ECG), and ECG-monitored step test or exercise testing has been practiced in Italy for over 30 years; this has facilitated the identification of cardiomyopathies and other cardiac diseases [2,3] and led to an 89% reduction in sudden cardiac death (SCD) [4]. It is pivotal to distinguish normal (i.e., training-related) ECG findings from borderlines and those classified as outright pathological (Figure 1) [5], Stein and Froelicher [6,7,8]. Here, we propose a practical guide for reporting the possible ECG findings that a cardiologist or sports physician may encounter in everyday clinical practice.

In particular, in Figure 1, ECG patterns are categorized into “electrical” and “structural” and are distinguished into normal, borderline, and abnormal by different colors.

## 2. Factors Influencing ECG Patterns in Athletes

During prolonged exercise, pressure and volume overload on the cardiovascular system leads to structural, functional, and electrical adaptations, collectively termed “athlete’s heart” aimed at improving O_2_ delivery to organs and working muscles. These adaptations include modifications in stroke volume (SV), heart rate (HR), blood pressure, peripheral vascular resistance, left ventricle (LV) afterload, cardiac chamber size, thickness and morphology, and sympathetic/parasympathetic balance [9,10,11,12,13,14,15].

The degree of remodeling is influenced by the intensity and type of discipline (static, dynamic, or mixed), environmental factors, and individual characteristics (i.e., sex, race, and age) [9,12,16,17].

As outlined in Table 1, electrical (increased vagal tone, intrinsic pacemaker cells, and AV node adaptations) and structural (eccentric and concentric changes) ECG adaptations are common among athletes. In addition, some ECG patterns, typically considered pathological in sedentary individuals, may be regarded as normal in athletes.

Sinus node adaptation is characterized by reduced sinus automaticity resulting from a resting increase in parasympathetic tone (efferent vagus nerve activity) and reduction in sympathetic input (decreased expression of the pacemaker current If) Stein and Froelicher [6,18]. In addition, possible structural changes (i.e., size, thickness, and morphology) may be part of the remodeling process, explaining why there is no complete reversibility of sinus bradycardia and sinus pauses after an athlete’s career cessation [18]. A thorough classification of sports disciplines considers four groups: skill, power, mixed, and endurance [14,15,19]. Endurance athletes have an increased vagal tone and demonstrate the highest increase in LV end-diastolic volume (i.e., eccentric remodeling), translating into enhanced SV at rest and during exercise [9,14,15]. Power athletes are characterized by concentric remodeling with accentuated ventricle wall thickness increase in response to bouts of power exercises [9,14,15].

Available data suggest that female players show less physiological remodeling than their male counterparts [20], even when cardiac dimensions are corrected for the typically smaller female body size. Moreover, adolescent athletes frequently show a typical ECG pattern consisting of T-wave inversion (TWI) in leads V1–V4, and TWI beyond V2 usually disappears after age 16 in Caucasians [21]. Furthermore, athletes of African and Afro-Caribbean origin (i.e., black athletes) tend to have thicker LV walls (up to 3% of them have a wall thickness >15 mm) than their white counterparts, and hence, a higher prevalence of abnormal ECGs [22,23]. In addition, black athletes exhibit more pronounced repolarization changes (TWI and more pronounced ST-segment elevation than their white counterparts) [24]. These differences were present independent of sex, as demonstrated in a female cohort [25].

The increase in cardiac chamber volume and thickness observed in athletes contrasts with that observed in cardiomyopathies [12]. However, extreme cases of exercise-induced remodeling may be difficult to differentiate from associated early cardiomyopathy, and other second-level methods can help discriminate the conditions [26].

In some selected cases, 3–6 months of detraining can be advantageous in documenting the significant reduction in some ECG patterns [27]. Another essential aspect to consider is the dynamic modification of ECG findings induced by a light exercise activity, such as jogging on the spot or climbing stairs [27]. Indeed, the autonomic nervous system modulates ECG patterns in athletes and varies with HR, since slowing HR exaggerates ST-segment elevation, whereas increasing HR reduces and can even eliminate early repolarization (ER) changes.

In addition, acute exercise induces hemodynamic changes that translate into electrocardiographic alterations, which are detectable immediately or shortly after the end of competition/training. P-wave voltage increase and duration, right atrial enlargement, increased R-wave amplitude in V1, and rightward shift in the QRS axis have all been observed after an ultramarathon race, possibly reflecting acute right-heart overload [28,29]. In addition, myocardial electrical instability may persist until the electrolytic imbalance is completely recovered [22]. Therefore, ECG for preparticipation screening purposes should be acquired at least the day after substantial and prolonged physical effort.

## 3. Normal, Borderline, and Abnormal Electrocardiographic Patterns in Athletes

The 2017 International Consensus Standards for ECG interpretation in athletes foresee three ECG findings: normal, borderline, and abnormal [7].

Up to 60% of athletes demonstrate ECG findings that are considered “normal” and result from autonomic modulation in the sinus node and structural changes [5,6]. Athletes’ most common expected ECG findings include sinus bradycardia, sinus arrhythmia, and sinus pauses. Other common ECG findings include increased QRS voltage, incomplete right bundle branch block (iRBB), ER, ectopic atrial rhythm, junctional escape rhythm, first-degree atrioventricular (AV) block, and second-degree AV block Mobitz type I [5,6]. Also, some repolarization variants are considered normal in black athletes (black repolarization pattern) and in athletes under 16 years (juvenile repolarization pattern) [5,6,7].

Some ECG patterns represent a gray zone that needs a proper differential diagnosis between normal and pathological [7]. These “borderline” patterns include right and left atrial enlargement, right and left-axis deviation, and complete right bundle branch block (CRBBB). These borderline findings in isolation likely represent physiological cardiac remodeling, particularly in athletes who are asymptomatic and have no family history of premature cardiac disease or SCD. Instead, the simultaneous presence of at least two borderline ECG patterns may underlie pathological cardiac disease, warranting additional testing [22,30].

Some definitively “abnormal” ECG findings may be the first manifestation of cardiomyopathy before morphological changes are present. Uncommon and training-unrelated ECG changes include TWI, ST-segment depression, pathological Q waves, left or right axis deviation, intraventricular conduction defects, ventricular pre-excitation, right ventricular hypertrophy (RVH), and Brugada-like repolarization changes [5]. These ECG findings alone do not necessarily imply a disease process, but indicate that more evaluation is needed. Therefore, some areas of uncertainty exist, as reported in Table 2.

## 4. Electrical Patterns

### 4.1. Sinus Bradycardia or First-Degree Atrioventricular Block

Resting sinus bradycardia, an HR < 60 beats per minute (bpm), is the most common bradyarrhythmia and is related to training level (occuring in up to 80% of highly trained endurance athletes) [7]. HR is lower in athletes than in their sedentary counterparts due to increased vagal tone. An HR between 30 and 60 bpm is benign unless associated with symptoms or other arrhythmias [6]. In addition, sinus bradycardia in a healthy athlete should resolve with the onset of physical activity [6]. However, even in asymptomatic athletes, resting bradycardia may represent a predisposing factor for atrial or ventricular extrasystole, and rarely for atrial fibrillation (AF) [18]. In addition, in some cases of severe sinus bradycardia, the sinus rhythm competes with the nodal rhythm, and isorhythmic AV dissociation can be observed (although ventricular escape rhythm is a rare finding) [6,7].

The effects of increasing age may modify some physiological adaptations at the sinus node, since resting bradycardia is more pronounced in older athletes whose maximum HR is also less, accounting in part for the drop-off in performance that occurs with age [6].

Although a resting HR ≤ 30 bpm, a PR interval ≥ 400 ms, or a sinus pause ≥ 3 s may be expected in a well-trained athlete, it should prompt further evaluation and need to be distinguished from sinus node disease [30]. Assessment of profound sinus bradycardia should include estimating the chronotropic response to a small amount of aerobic exercise. Indeed, further testing should be performed if the HR does not increase or the PR interval does not normalize appropriately, particularly if the athlete experiences symptoms or has a family history of cardiac disease or SCD [7,30].

Notably, the prevalence of first-degree and Mobitz type I second-degree AV blocks (Luciani-Wenckebach) is very high in endurance athletes. In contrast, higher AV block degrees (Mobitz type 2 second- or third-degree AV block) are sporadic [31] and need further assessment [32].

### 4.2. Sinus Arrhythmia

It is defined as excessive HR variation with respiration (increase during inspiration and decrease during expiration) [30], resulting in an irregular heart rhythm originating from the sinus node [10].

It is the consequence of the physiological adaptation of the HR to respiratory phases [10], originating because the pulmonary center in the brainstem blocks vagal activity in the lungs during inspiration, determining bronchodilation and increasing the HR [6]. It is also possible that atrial stretch receptors are involved, which respond to variations in venous return by increasing the HR during inspiration or slowing the HR during expiration [6].

Sinus arrhythmia represents a physiological marker of a healthy and well-functioning heart and is frequently exaggerated in children and young adults [6]; it is also more prevalent in trained athletes (up to 55%) than in the general population [31]. A more pronounced sinus bradycardia is typically associated with more obvious sinus arrhythmia. Only profound sinus bradycardia or marked sinus arrhythmia (i.e., HR less than 30 bpm or pauses of 3s during waking hours) require further assessment and must be distinguished from sinus node dysfunction (e.g., in sick sinus syndrome) [6,7,10]. The P-wave axis in the frontal plane remains normal in sinus arrhythmia, and the fluctuation in HR should resolve with the onset of exercise [7]. Conversely, features suggesting sinus node dysfunction include a lack of rhythmic changes in the HR, abrupt sustained HR increases and decreases, prolonged pauses or periods of sinus arrest, and an inappropriate response to exercise (slowed acceleration, inappropriately rapid deceleration, or presence of symptoms) [7].

If sinus arrhythmia is present with beat-to-beat variation in the HR, an average QT interval and RR interval should be used [30]. In addition, sinus arrhythmia can cause a partly irregular rhythm during the auscultation of heart sounds.

### 4.3. Sinus Pauses

Sinus pauses exceeding 3 s are very common among athletes due to intrinsic electrophysiological adaptation or intense parasympathetic tone. These phenomena may be expected to be rhythm variants in well-trained individuals and occur with slow resting HRs, typically at rest and during sleep, and can sometimes result in impressive and prolonged sinus node arrest (>6 s) [6]. However, invasive electrophysiological studies typically demonstrate normal sinus node and AV node functions, and a normal sinus rhythm appears during exercise [6].

Viitasalo et al. [33] reported sinus pauses exceeding 2.0 s at Holter monitoring in 37.1% of young, highly trained endurance athletes. In sedentary subjects, sinus pauses are considered pathological when they are >3 s. However, when isolated and not associated with symptoms or arrhythmias, this threshold should not be considered a sign of sinus node dysfunction in aerobic athletes [34]. A 5 s pause provided greater specificity but lacked sensitivity for detecting symptomatic patients [34]. Distinguishing these cardiac pauses, which are worrisome and merit treatment, from more benign forms is not straightforward [34]. However, high-grade AV block during exercise, chronotropic incompetence, or strain-induced pauses (e.g., during weightlifting) are abnormal and may signal an underlying disease [34].

### 4.4. Junctional Escape Rhythm

In a junctional escape (nodal) rhythm, the QRS rate is faster than the sinus node activity; the QRS complex is narrow (<120 ms unless the baseline QRS complex is conducted with aberrancy, i.e., already large due to a bundle branch block), the R-R interval is regular, and the HR is typically <100 bpm [6,7]. A junctional escape rhythm or a wandering escape pacemaker occurs with a frequency of up to 8% in all athletes under resting conditions, and a sinus rhythm should resume by increasing HR, like with physical activity [6,7].

In isorhythmic dissociation (i.e., AV dissociation without block), the junctional pacemaker is faster than the sinus node, and the ECG trace shows more QRS complexes than P waves (intermittent ventricular capture by sinus P waves, resulting in an irregular ventricular response) [6,7]. It is not pathological since it expresses an autonomic mismatch between the AV and sinus nodal modulation [7]. However, it must be differentiated from a complete AV block, where the atria and ventricles are independent.

### 4.5. Ectopic Atrial Rhythm

An ectopic atrial rhythm in an athlete occurs because of a slowed resting sinus rate due to intrinsic electrophysiological adaptation or increased vagal tone [6,7]. It is identified when P waves have different morphologies, with an atrial rate typically <100 bpm. The normal origin of the cardiac rhythm is the sinus node, which manifests on ECG with the P-wave axis between 0° and 90°. The most easily encountered finding is an ectopic P wave in the lower right atrium (i.e., a coronary sinus rhythm), which is considered normal during childhood with no need for cardiac assessment. A wandering atrial pacemaker (more than two types of P-wave morphology detection) is also common. Normal P-wave morphology should resume with the onset of physical activity [6]. Other P-wave origins, atrial enlargement, and hypertrophy should be investigated to exclude heart disease [32].

### 4.6. First-Degree AV Block (PR Interval up to 399 ms)

The PR interval is measured from the beginning of the P wave to the Q wave or the R wave if no Q wave is present. In the first-degree AV block, the PR interval is >200 ms but presents the same duration on every beat [6]. The PR interval increases with age and decreases with HR.

Hiss and Lamb reported that the incidence of first-degree AV block was approximately 0.65/1000 in a cohort of more than 120,000 young subjects, mainly aviators [35]. A mild-to-moderate first-degree AV block (PR interval between 200 and 399 ms) is frequently found in aerobic athletes on resting ECG, representing a delay in AV nodal conduction due to increased vagal drive or intrinsic AV node adaptation [6]. It is benign and does not need a further cardiological evaluation if the PR shortens appropriately at higher HRs (with exercise or hyperventilation) [6,7,32,36,37,38]. A further evaluation should be performed if the PR interval does not resolve, depending on the clinical scenario (symptoms of syncope or a family history of cardiac disease or SCD) [7,38]. When the PR interval is markedly prolonged, especially in young athletes, autoimmune-mediated forms of AV block due to maternal circulating anti-Ro/SSA-antibodies should also be excluded [39]. Generally, a PR interval of ≥ 400 ms is considered significantly prolonged and requires further evaluation [7].

### 4.7. Mobitz Type I Second-Degree AV Block

A Mobitz type I second-degree AV block is characterized by the progressive (from beat to beat) PR interval lengthening until a P wave is non-conducted. It may be present in up to 10% of athletes, and characteristically, normal AV conduction immediately resolves with faster HRs during exercise [37]. This bradyarrhythmia should be considered benign in well-trained athletes with an appropriate chronotropic response and without symptoms [10].

### 4.8. High-Grade Atrioventricular Block

Mobitz Type II second-degree AV block and third-degree (i.e., complete) AV block are not expected features in conditioned athletes and should be considered abnormal. Mobitz Type II AV block is characterized by intermittently non-conducted P waves without progressive prolongation of the PR interval (the PR interval in the conducted beats remains constant), and the RR interval surrounding the dropped beat(s) is an exact multiple of the preceding RR interval. In a complete AV block, there is severe bradycardia due to the absence of AV conduction and AV dissociation, with independent atrial and ventricular rates; in this case, the ventricular rhythm is usually maintained by a junctional or ventricular escape rhythm.

Mobitz II AV block and complete AV block can mask underlying cardiac disease, so athletes require further diagnostic investigations, and referral to an electrophysiologist is usually advisable [30].

### 4.9. Pre-Excitation

**Figure 2:**
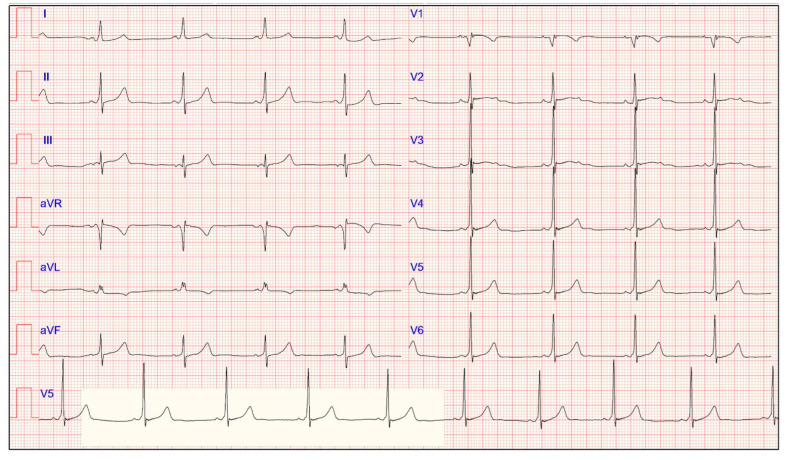
Electrocardiogram of a 29-year-old asymptomatic male athlete displaying typical ECG features of ventricular pre-excitation, characterized by a short PR interval and slurring of the initial segment of the QRS complex (delta wave). These findings are due to a right anterior accessory pathway, as indicated by the negative delta wave in lead V1 and the positive delta wave in the inferior leads and V3.

Ventricular pre-excitation occurs in the presence of an accessory pathway that bypasses the AV node, leading to abnormal electrical conduction to the ventricle [7]. Typical ECG features of ventricular pre-excitation in the Wolff-Parkinson-White (WPW) syndrome include a PR interval (<120 ms) with slurring of the initial segment of the QRS complex (the “delta wave”) and a widening of the QRS complex with a total duration >120 ms (Figure 2) [27]. The delta wave may be particularly evident in highly trained athletes since they may show increased vagal tone and AV node conduction time. The WPW pattern occurs in approximately 1/1000 to 4/1000 athletes [7]. It may be genetically inherited in 2–4% of cases, and it may also be associated with conditions like an Ebstein’s anomaly or syndromic hypertrophic cardiomyopathy (HCM) caused by glycogen storage disorders [27]. Different types of tachyarrhythmia may complicate the WPW syndrome; for example, AV re-entry tachycardia (AVRT) and pre-excited AF can both degenerate into potentially fatal ventricular fibrillation.

A short PR interval in isolation in asymptomatic athletes without a widened QRS or delta wave should not be considered in further assessments. In contrast, asymptomatic athletes with WPW patterns should be further investigated to identify low-risk or high-risk accessory pathways [7]. The exercise stress test is the first test to be performed, in which a sudden and complete loss of pre-excitation at higher HRs may underline an accessory pathway with low risk. Invasive electrophysiology testing should be considered to determine the shortest pre-excited RR interval during AF when a non-invasive test is positive or inconclusive. Young athletes with the shortest pre-excited RR interval of ≤250 ms should proceed with transcatheter pathway ablation since high catecholamine concentrations during strenuous exercise may alter the refractory period of an accessory pathway, as in the case of symptoms or high-risk accessory pathways [7]. Sports eligibility may be allowed for athletes with no history of arrhythmias and whether the electrophysiology study demonstrates a refractory period of the accessory pathway ≥250 ms at rest and ≥210 ms during exercise or isoproterenol infusion [15].

### 4.10. Long QT Interval

Long QT Syndrome (LQTS) is a rare genetic condition caused by mutations in cardiac ion channels and is associated with ventricular arrhythmias (VAs) and SCD. The most common forms are types 1, 2, and 3 LQTS due to mutations in the genes KCNQ1, KCNH2, and SCN5A, respectively.

LQTS is diagnosed when QTc is ≥480 ms on repeated 12-lead ECGs with or without symptoms or when the LQTS diagnostic score is at least 3. The QT interval is measured using the lead with the longest interval across multiple cardiac cycles. In well-trained athletes, U waves are typically observed as positive deflections following the T wave. However, the appearance of large or negative U waves, particularly in the lateral leads, may be indicative of underlying pathological conditions. When evaluating the QT interval, large U waves fused with the T wave are included, while smaller or separate U waves are excluded. The end of the T wave is determined using the maximum slope-intercept method. For notched T waves, the interval ends where the isoelectric line intersects the tangent of the second notch’s steepest downward slope. A diagnosis is also made when a pathogenic mutation is present, regardless of the QT duration [40]. LQTS should also be considered in cases where QTc is between 460 ms and 480 ms on repeated 12-lead ECGs in patients with arrhythmic syncope, provided that secondary causes of QT prolongation, such as hypokalemia and QT-prolonging drugs, have been excluded. In addition, athletes should be evaluated more than 48 h after endurance exercise, which has been associated with QT prolongation [41].

Basavarajaiah et al., in 2000 elite athletes with a mean age of 20 years, found seven athletes with a QTc interval of 460–570 ms. In those with a QTc interval ≥ 500 ms, there were other signs of LQTS (paradoxical prolongation of the QTc during exercise, gene mutation, and QTc prolongation in first-degree relatives) [23]. Therefore, an athlete with a QTc ≥ 500 ms and no reversible causes should be promptly referred to an electrophysiologist [42]. Instead, the presence of a QTc between 470 and 500 ms in male athletes and 480–500 ms in female athletes constitutes a ‘gray zone’ of high suspicion of LQTS, so an ECG should be repeated on a different day, and the personal and familial history should be investigated appropriately. However, if QTc is <500 ms without symptoms or familial disease, a diagnosis of LQTS is unlikely [23].

In addition, some authors discovered that among athletes lacking the specified genotype, over 40% experienced complete or near-complete normalization of their ECGs following a 3-to 4-month detraining period [43].

### 4.11. Short QT Interval 

Short QT syndrome (SQTS) is a rare and potentially lethal channelopathy. In the most recent guideline document, two QTc cut-off thresholds have been proposed for diagnosis: a QTc ≤ 320 ms alone or a QTc ≤ 360 ms combined with a family history of SQTS, aborted cardiac arrest in the absence of heart disease, or pathogenic mutation [44,45]. Dhutia et al. [46] showed an excellent prognosis among young individuals with QTc < 320 ms since none of these subjects experienced arrhythmias, syncope, or SCD. However, a short QT interval in the setting of SCD has been associated with a risk of recurrence. Therefore, athletes with suspected SQTS and a high risk for cardiac events should be further investigated and restricted from participation in sports during evaluation.

### 4.12. Brugada Type 1 Pattern

Brugada type 1 pattern is an inherited primary electrical disease defined as an rSr′ pattern with coved ST elevation ≥ 0.2 mV (2 mm) and inversion of the terminal segment of the T-wave in leads V1–V3. The type 1 pattern is the only ECG diagnostic pattern for Brugada Syndrome (BrS), which predisposes to VAs and SCD, typically during rest or sleep. Brugada type 2 is a suggestive rather than a diagnostic pattern characterized by a saddleback-shaped ST elevation of at least 2 mm. Finally, Brugada type 3 can exhibit the morphology of either type 1 or type 2, but with less than 2 mm of ST-segment elevation.

In an athlete’s ECG, the Brugada type 1 pattern must be distinguished from the ST-segment elevation of the ER, which is generally benign. In this context, the “Corrado index” can provide more information by evaluating the ST-segment elevation at the start of the ST-segment/J-point and 80 ms later [47]. A ratio greater than 1 indicates a Brugada pattern. If the athlete’s ECG shows a type 1 Brugada pattern, evaluation by an electrophysiologist is suggested, regardless of symptoms or arrhythmias. If the ECG pattern is not better defined, an ECG with high precordial leads (i.e., leads V1 and V2 placed in the second or third intercostal space) should be performed; if this unmasks a Brugada pattern type 1, the athlete needs an electrophysiologist evaluation [24].

### 4.13. Multiple Premature Ventricular Contractions

**Figure 3:**
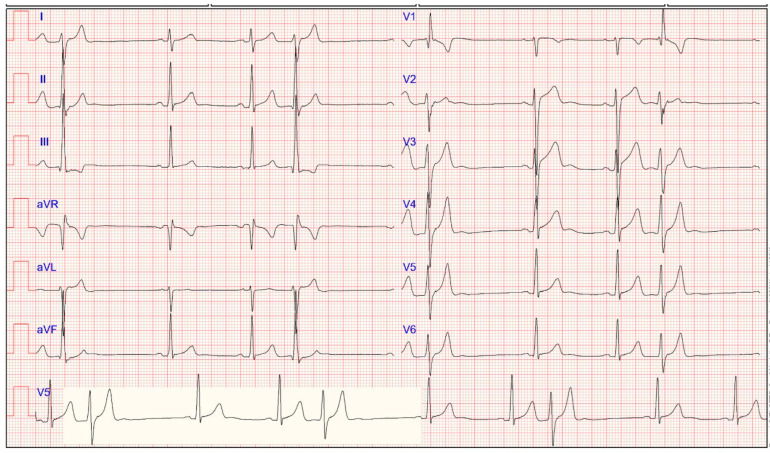
Electrocardiogram of an 18-year-old asymptomatic female athlete showing PVCs, including occurrences of ventricular bigeminy.

Premature ventricular contractions (PVCs) are premature beats arising from an ectopic focus within the ventricles, and are common in athletes [48]. Although multiple PVCs are usually benign, their presence may be a hallmark of underlying heart disease at risk of SCD. Hence, it is essential to differentiate PVCs with a low probability of an underlying disease from those associated with structural substrates (Figure 3).

The first element to consider is the arrhythmic burden, i.e., the number of PVCs in 24 h and more complex arrhythmias (couplets, triplets, or runs of non-sustained ventricular tachycardia). According to International criteria, further investigations are needed when two or more PVCs are observed on a standard (10-s) single ECG tracing because they suggest a high 24-h PVC burden and are considered abnormal. Recently, Zorzi et al. demonstrated that there is no solid evidence supporting the hypothesis that endurance sports activity increases the PVC burden [49].

Multiple (≥2) PVCs are uncommon and are present in <1% of 12-lead ECGs in athletes [30]. However, there is variability throughout the day or day-to-day in the PVC burden [50]. Biffi et al., among athletes with ≥2000 PVCs per 24-h, up to 30% were found to have underlying structural heart disease, compared with 3% in those with <2000 PVCs per 24-h [51].

PVC morphology may play a role in differentiating healthy athletes from those with cardiac disease. PVCs found in athletes usually have a morphology indicating RV or LV outlet origin (with left bundle branch block (LBBB) inferior axis and R/S transition beyond V3 and V1–V2, respectively) or fascicular origin (RBBB and relative narrow QRS). On the contrary, different morphologies like LBBB and intermediate/superior axis or RBBB and large QRS are rare in athletes and can indicate an underlying cardiac disease. PVCs originating from the right ventricular outflow tract are considered remarkably benign when associated with a normal ECG; however, this PVC morphology can also be present in patients with early ARVC, particularly when the QRS exceeds 160 ms [52]. Nevertheless, patients with ARVC usually show PVCs with LBBB morphology and a superiorly directed axis, which suggests an origin from the non-outflow tract portion of the right ventricle (RV) [52]. In addition, ECG abnormalities associated with ACM include repolarization abnormalities, such as negative T-waves and ST-T segment depression, pathological Q waves, conduction disturbances, ventricular pre-excitation, long and short QT intervals, and Brugada-type repolarization abnormalities [53].

Other features that increase or decrease the probability of an underlying condition include whether PVCs are monomorphic or not, the presence of complex patterns (couplets with short RR intervals, non-sustained ventricular tachycardia, “R on T” phenomenon), reproducibility on repeated exercise testing, or 24 h ambulatory ECG monitoring (24 h-ECG) including a training session and their behavior during exercise. Indeed, PVCs suppression with exercise is typical of benign forms (RV outlet PVCs usually show this pattern) [54]. On the contrary, PVCs that start or continue with an ECG stress test should be carefully considered, especially if they are of unusual morphology and require further investigation, especially CMR, to provide an accurate assessment of chamber dimensions and function, and accurate tissue characterization [55].

Moreover, the ECG abnormalities associated with severe cardiac disease include repolarization abnormalities such as negative T-waves and ST-T segment depression, pathological Q waves, conduction disturbances, ventricular pre-excitation, long and short QT intervals, and Brugada-type repolarization abnormalities [53].

Following this initial assessment, most athletes can be assured that PVCs are likely benign, allowing them to be cleared for competition with regular follow-up. However, further investigations should be carried out when clinical suspicion is high [48].

### 4.14. Ventricular Arrhythmias 

Awareness of VAs in athletes has increased as recent data have suggested that strenuous physical activity might promote a substrate for potentially lethal arrhythmia occurrence in otherwise healthy individuals. This phenomenon raises the question of how much sport may trigger arrhythmias in some athletes and whether it is necessary to limit physical activity to prevent them [56].

Athletes can experience a variety of VAs caused by structural or electrical diseases, including idiopathic causes, cardiomyopathies, ion channelopathies, myocarditis, myocardial infarction, or sarcoidosis. Generally, the more frequent and complex the VAs (couplets, triplets, or VT), the higher the risk of underlying cardiomyopathy. The prevalence of VAs largely depends on individual factors, such as age, comorbidities, and duration of recording [57]. As in the general population, athletes with VAs may be asymptomatic or present with symptoms like palpitations, a decline in physical fitness, dizziness, or syncope. Approximately 15% of athletes with persistent, frequent VA will present progressive LV systolic dysfunction (i.e., VA-induced cardiomyopathy) or result in sustained VT and SCD [58]. A detailed family history, echocardiogram to check for structural heart problems, CMR to look for cardiomyopathies, ambulatory ECG monitoring, and exercise ECG testing should be performed. A further evaluation with an electrophysiology study or genetic testing may be required [7].

### 4.15. Atrial Tachyarrhythmias

Athletes can suffer from several kinds of atrial arrhythmias. Sinus tachycardia is the most common atrial tachyarrhythmia and is rarely caused by intrinsic cardiac disease. Potential causes include recent exercise, anxiety, fever, infection, dehydration, stimulant use, anemia, hyperthyroidism, or, in rare cases, underlying cardiac or pulmonary conditions.

Sports and exercise training are not associated with a higher risk of supraventricular tachycardia (SVT), and its prevalence is comparable to that in the general population [59]. Atrial tachyarrhythmias are rarely fatal but can be associated with other conditions that can lead to SCD, including LQTS, WPW, BrS, myocarditis, congenital heart disease, and cardiomyopathies.

AVNRT is the most prevalent rhythm disturbance; orthodromic AVRT with retrograde conduction over an accessory pathway or ectopic AT can also be experienced. When the accessory pathway can conduct anterogradely from the atria to ventricles during sinus rhythm, it is possible to visualize ventricular pre-excitation on ECG. In this case, the patient with paroxysmal SVT had WPW syndrome, which carries a risk of SCD and, therefore, should be referred to an expert electrophysiologist.

In the case of paroxysmal SVT, a repeat ECG should be obtained when the patient is not in SVT. If the Valsalva maneuver, carotid sinus massage, or diving reflex terminates arrhythmia, obtaining a rhythm strip for a detailed analysis of the SVT mechanism is advisable. Additional diagnostic steps include an echocardiogram, ambulatory ECG monitor, and exercise treadmill test. Consideration for referral to an electrophysiologist may be justified for an electrophysiology study and potential ablation.

Athletes have an approximately five-fold increased lifetime risk of AF compared to sedentary individuals, despite a lower prevalence of conventional AF risk factors. Light-to-moderate physical activity is associated with a significantly lower risk of AF, while vigorous physical activity, especially endurance, increases the risk of AF [60]. Risk factors for AF in athletes include male sex, middle age, endurance sports, tall stature, and a total lifetime exercise dose exceeding 1500–2000 h. The specific pathophysiology of AF in athletes is not fully understood, but shortening of the atrial refractory period, atrial stretching, atrial inflammation, and scarring are involved [61].

Cavigli et al. analyzed the acute effects of an ultramarathon race in 68 healthy master athletes. They demonstrated the absence of biatrial functional remodeling as a substrate for supraventricular arrhythmias, countering the hypothesis of acute mechanical biatrial dysfunction induced by ultra-endurance exercise [62].

Atrial Flutter (AFL) is usually due to the macro re-entrant circuit around the tricuspid valve; it may coexist with AF or develop after administering class I antiarrhythmic drugs for AF. If AF or AFL is found, an echocardiogram should be completed to assess for structural heart disease, and anti-coagulation should be considered based on standard guidelines [63]. An ambulatory ECG monitor should be used to assess whether the rhythm is paroxysmal or persistent and what the ventricular rate is throughout the day. Cardiac MRI, electrophysiology study with possible ablation, or genetic testing may be considered [30]. Patients with a history of AF or AFL must avoid contact with sports and stop physical activity when palpitations or other significant symptoms occur.

## 5. Structural Patterns

### 5.1. Axis Deviation and Atrial Enlargement

A left-axis deviation is defined as an electrical axis ranging from −30° to −90°, while the right axis deviation is an axis > 120°. A left atrial enlargement is characterized by a prolonged P wave duration exceeding 120 ms in leads I or II, accompanied by a negative portion of the P wave with a depth of at least 1 mm and a duration of at least 40 ms in lead V1. Instead, right atrial enlargement is a P wave ≥ 2.5 mm in II, III, or aVF.

An isolated axis deviation is present in 2% of healthy athletes [27]. Gati et al. [64] investigated 2533 young athletes with ECG and echocardiography, reporting that isolated axis deviation and atrial enlargement prevalence were slightly higher compared with controls (5.5 vs. 4.4% respectively); however, when isolated, these alterations do not seem to indicate an underlying cardiac disease.

### 5.2. Incomplete Right Bundle Branch Block

An iRBBB, i.e., a QRS duration < 120 ms, is also a prevalent finding in athletes, with a frequency of 35–50% compared to less than 10% in the general population [53]. Notably, the presence of iRBBB is related to years of training [65], occurs mainly in those engaged in endurance training and mixed sports disciplines, and is reversible with deconditioning [53]. An iRBBB may be related to RV dilation and remodeling (relative reduction in the RV systolic function at rest and interventricular dyssynchrony), resulting in increased conduction time and not by an intrinsic delay within the His-Purkinje system [10].

An iRBBB in an asymptomatic athlete with no additional pathological findings (no family history or normal physical examination) does not prompt further evaluation. However, particular attention should be paid to the concomitant presence of ST-T abnormalities. Indeed, a typical iRBBB is uncommon in ARVC; however, an iRBBB with TWI in the mid-precordial leads beyond V2, low-limb lead voltages, and PVCs with an LBBB pattern may be present in ARVC. Moreover, an iRBBB should not be confused with the Brugada ECG pattern, which typically includes a high takeoff and downsloping ST-segment elevation followed by a negative T-wave in ≥2 leads in V1–V3 [66].

### 5.3. Complete Right Bundle Branch Block

A CRBBB is defined as QRS duration > 120 ms, an rSR′ pattern in lead V1, and an S wave wider than the R wave in lead V6 [7]. CRBBB is detected in almost 1% of the general population, and its prevalence in young adult athletes is about 0.5–2.5%. This may be related to the structural remodeling characterized by RV dilation with QRS widening and decreased RV systolic function in trained athletes.

Among 510 US athletes, those with RBBB had larger LV dimensions and a reduced ejection fraction, but the fractional area change was preserved. However, they did not display any evidence of pathology [1,67,68].

Incidentally, the prevalence of cRBBB is lower than that of iRBBB. Right ventricular remodeling during strenuous exercise has been associated with iRBBB. In contrast, the significance of a cRBBB is less expected and may represent a more extreme right ventricular adaptation to exercise. Based on these considerations, cRBBB is considered a borderline variant in athletes, and transthoracic echocardiography may discriminate against underlying heart disease [67,68].

### 5.4. Left Bundle Branch Block

LBBB is defined as a QRS duration > 120 ms and a specific pattern (dominant S wave in V1, broad monophasic R wave in lateral leads, absence of Q waves in lateral leads, and prolonged R wave peak time > 60 ms in leads V5–V6). Its prevalence in athletes is about 0.1% but is frequent in patients with cardiomyopathy and ischemic heart disease [68,69,70,71,72].

Hence, LBBB should always be considered abnormal; athletes with this finding necessitate cardiological workup, including transthoracic echocardiography, Holter monitoring, and second-level investigations like a CMR or coronary CT angiography. Little is known about the pathogenesis and prognostic significance of LBBB in young adults without heart disease. Several possible causes may be hypothesized, like familial genetic diseases involving ion channels (Lenègre disease), concealed coronary artery disease, primary dilated cardiomyopathy, and myocarditis. [14]. Finally, some authors [73]. have suggested that LBBB may cause induced cardiomyopathy by creating an asynchrony of contraction of the LV.

### 5.5. Profound Non-Specific Intraventricular Conduction Delay

Non-specific intraventricular conduction delay (NICD) is defined as a QRS duration of >110 ms without a specific morphology. It is usually reported in the general population but is also documented among patients with cardiomyopathy and has been associated with a higher risk of cardiovascular death [74]. The significance of NICD with normal QRS morphology in healthy, asymptomatic athletes is unclear [75] and is possibly due to neurally mediated conduction fiber slowing and increased myocardial mass [76].

The exact cut-off to prompt further investigation in athletes with an NICD has yet to be established. No further diagnostic evaluation is required for asymptomatic athletes with isolated NICD < 140 ms. However, athletes with cardiovascular symptoms, a stated family history of sudden death, or presumed cardiomyopathy with an NICD < 140 ms, or in combination with other abnormal findings on surface ECG, should be investigated. Evidence suggests that marked NICD ≥ 140 ms, regardless of QRS morphology, is abnormal and should be evaluated with an echocardiogram as the first-line examination and other tests depending on clinical suspicion [7].

### 5.6. Early Repolarization

**Figure 4:**
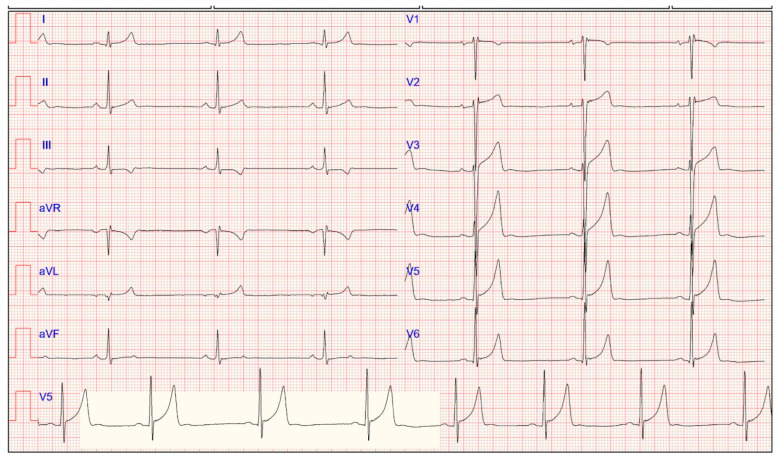
Electrocardiogram of a 24-year-old asymptomatic female athlete displaying typical ECG features of early repolarization, characterized by J-point elevation, which is more evident in precordial leads.

ER, also known as “J-waves” or “J-point elevation”, is an electrocardiographic phenomenon consistent with the elevation in 2 contiguous leads of the J-point (a bland transition from the QRS segment to the ST-segment) [6]. J-point elevation can manifest as either QRS slurring (transition from the QRS segment to the ST-segment) or notching (positive deflection inscribed on the terminal S wave). It can appear in any lead but is most likely present in the anterior leads (Figure 4).

ER is commonly found in healthy individuals (2–44%) but is even higher (50–90%) in athletes, particularly those of young, male, and black ethnicity [7,66,77,78].

The most common pattern in Caucasians consists of J-point elevation, with an upward concave ST-segment displacement ending in a positively peaked and tall T-wave. These changes are more marked in V3–V4; however, maximal ST-segment elevation may also be seen in the lateral (V5, V6, I, and aVL) or inferior (II, III, and aVF) leads [6,66].

ER can present markedly different patterns in athletes of Afro-Caribbean descent (i.e., black athletes). A common repolarization variant in this population includes an elevated J-point and ST-segment with upward convexity, followed by asymmetric TWI in the anterior leads (V2–V4) [7]. More than two-thirds of black athletes exhibit ST-segment elevation, and up to 25% show TWI [79,80]. Therefore, TWI in leads V1–V4, when preceded by J-point elevation and convex ST-segment elevation, should be considered a normal variant as part of the ‘black athlete’s heart’ [79]. No further investigation is necessary if there are no additional clinical or ECG features suggestive of cardiomyopathy [7,24].

Specific morphological features assist clinicians in distinguishing the ER pattern common in athletes (referred to here as a “benign pattern”) from the pattern observed in patients at risk of ventricular fibrillation (the so-called “malignant pattern”). In black Afro-Caribbean athletes, normal repolarization changes do not extend beyond V4 [10,66]. Conversely, a horizontal or descending ST-segment elevation following J-point elevation, as well as a QRS slurring or notching elevation ≥ 2 mm (0.2 mV), mainly localized in the inferior or inferolateral leads, has been associated with a higher risk of SCD (the so-called “malignant pattern”).

Nevertheless, currently available data are too scarce to assume a cause-effect relationship between the ER pattern and the risk of malignant VAs, so there is no way to know who would be at considerable risk of SCD [66]. Indeed, some reports have shown that in a medium-term follow-up period, the ER pattern in athletes does not convey any risk of adverse cardiac events, including SCD or ventricular tachyarrhythmias [81]. However, in athletes with syncope or resuscitated cardiac arrest, unexplained even after a detailed cardiological and neurological workup, the ECG pattern of ER in the inferior or lateral leads, particularly when associated with prominent terminal QRS slurring, should raise suspicion of underlying diseases or idiopathic ventricular fibrillation [53,66].

Because the autonomic nervous system modulates the ER pattern, it is exaggerated by a lower HR (i.e., increased vagal tone); in contrast, it is reversed and can even disappear with sinus tachycardia or deconditioning [66].

In middle-aged non-athletic Finnish citizens, QRS slurring or notching with horizontal ST-segment elevation in the inferolateral leads was related to an increased risk of arrhythmic death [82]. However, a high percentage of young competitive athletes (25–30%) show ER with similar morphological features in the inferior or lateral leads [83], a finding related to increased vagal tone.

### 5.7. ST-Segment Depression

ST-segment depression is a depression ≥0.5 mm (0.05 mV) measured in the J-60 or J-80 point in two or more contiguous leads. It is very rarely found in healthy athletes, but rather a common finding in cardiomyopathies, generally associated with additional ECG alterations, such as pathologic Q waves or inverted T waves. Therefore, demonstration of ST-segment depression on resting ECG should prompt a careful evaluation to exclude heart disease like HCM, DCM, LVNC, ARVC, myocarditis, or CAD [7,24,31,50,66].

### 5.8. Abnormal T-Wave Inversion 

Abnormal TWI indicates the presence of an inverted T-wave with a depth ≥1 mm in two or more contiguous leads, except V1, aVR, and DIII [24]. Abnormal TWI in an athlete’s ECG requires special attention because it can signal the underlying cardiomyopathies.

TWI can be classified as anterior (V2–V4), lateral (I, aVL, V5, V6), inferior (II, III, aVF), or inferolateral (I, II, aVL, aVF, V5–V6). Among these, lateral and inferior TWI are more frequently related to structural abnormalities of the heart.

Migliore et al. reported a prevalence of TWI of 5.7% among 2765 children aged 8–18 years who underwent preparticipation screening [84]. Most TWIs (4.7%) were localized to the right precordial leads (V1, V2, and V3) and were considered a juvenile pattern.

### 5.9. Anterior T-Wave Inversion 

An anterior TWI is present in up to 11% of healthy adolescent athletes [84] and is considered a typical finding in athletes aged <16 years without symptoms or family history [21] and in asymptomatic black athletes if preceded by J-point and ST-segment elevation [79].

The term “juvenile repolarization pattern” is defined by TWI V1–V3 in athletes < age 16 (i.e., who have yet to reach physical maturity) [84]. RV dominance and predominant posteriorly directed repolarization may explain this phenomenon in young teenagers. Anterior TWIs gradually resolve and eventually disappear as the child ages, with only 0.2% manifesting anterior TWI beyond V2 when aged ≥16 years [27]. However, it is difficult to provide a precise age cut-off to differentiate between the benign ‘juvenile pattern of repolarization’ and anterior repolarization abnormalities, which may represent the sign of underlying cardiomyopathy. Anterior TWI is rarely observed (2–4%) in patients with HCM, who more often show TWI in inferolateral leads [79]. However, it may be present in as many as 80% of patients with ACM [31].

Beyond the normal variants of anterior TWI, some elements support the ACM hypothesis, such as an epsilon wave, prolonged S wave upstroke in leads V1–V3, numerous PVCs, and low amplitude limb lead voltages [24]. The diagnosis requires additional examinations like echocardiography, CMR, Holter ECG monitoring, exercise ECG, and genetic testing.

### 5.10. Lateral and Inferior T-Wave Inversion

Lateral and inferior TWI are observed in various pathological conditions, including myocardial ischemia and infarction, bundle branch block, ventricular hypertrophy, pulmonary embolism, and HCM [85]. Therefore, second-level assessments, mainly cardiac MRI with late gadolinium enhancement, are necessary to exclude underlying cardiomyopathy.

A study evaluating repolarization changes in 904 black athletes indicated that anterior TWIs represent an ethnic physiological response to exercise. In contrast, athletes who exhibited TWIs in the lateral leads or ST-segment depression were more likely to show initial or incomplete expression of HCM [79], indicating that TWI in the lateral leads is always considered abnormal and requires additional testing to rule out HCM or other cardiomyopathies [10].

The TWI in inferior leads can be seen in HCM, although more rarely compared to lateral TWI. In addition, the significance of TWI in the inferior leads is probably a normal variant in black athletes.

### 5.11. Pathological Q Waves

A pathological Q wave implies a Q/R ratio ≥ 0.25, or a Q wave duration ≥ 40 ms in two or more contiguous leads, excluding DIII and aVR [24]. Pathological Q waves are described in about 1–2% of athletes and may be higher in black and male athletes [86]. Beyond false positive cases (uncorrected placement of ECG leads, e.g., V1 and V2 in higher intercostal spaces) [87], pathological Q waves have been described in some cardiomyopathies prior to myocardial infarction and WPW [24]. Accordingly, if pathological Q waves are found on an athlete’s ECG, particularly with other ECG abnormalities, an echocardiogram and eventually a CMR should be considered in cases of high suspicion of cardiomyopathy. Moreover, in athletes aged >30 years with cardiovascular risk factors, echocardiogram and inducible ischemia tests should be used to exclude ischemic heart disease [24].

### 5.12. Right Ventricular Hypertrophy

RVH evaluated by the voltage criterion is also common in athletes, with up to 13% fulfilling the Sokolow-Lyon criteria (RV1 + SV5 or SV6 > 1.05 mV) [79], representing the typical spectrum of physiological cardiac adaptations. Zaidi et al. found that the prevalence of the Sokolow-Lyon voltage criteria for RVH correlated poorly with increased RV wall thickness on echocardiography and that no patients with ARVC or pulmonary hypertension (PH) exhibited the voltage criteria for RVH without additional ECG abnormalities. Thus, RVH diagnosed using voltage criteria in asymptomatic young individuals with no additional pathological findings (negative family history and normal physical examination) does not prompt further evaluation. In contrast, particular attention should be paid to the co-existence of pathological Q waves, TWI, axis deviation, ST-segment depression, or atrial enlargement because ARVC and PH can rarely be present with RVH voltage criteria [88].

### 5.13. High and Low QRS Voltages

Cardiac remodeling in athletes is associated with increased chamber dimensions and left ventricular wall thickness and mass. Indeed, a physiological QRS increase is widespread in athletes (up to 45%), particularly in males, the black Afro-Caribbean, and those engaged in endurance sports. A physiological QRS increase is associated with a normal QRS axis, atrial and ventricular activation patterns, and ST-segment and T-wave repolarization.

Further evaluation should only be considered in the presence of other non-voltage criteria for LV hypertrophy, like atrial enlargement, left-axis deviation, ST depression, pathological Q waves, or TWIs [66]. Indeed, these patterns are not usually seen in athletes and should raise suspicion of underlying pathologies, such as aortic valve disease, hypertension, or HCM. Although HCM can present with an isolated increased QRS voltage, this is uncommon [66,89].

**Figure 5:**
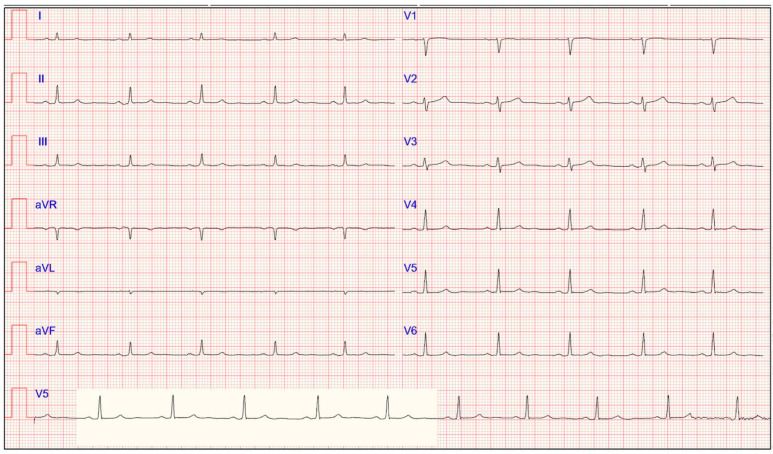
Electrocardiogram of a 26-year-old male athlete with non-cardiac pathological conditions showing common ECG features of low QRS voltages, characterized by QRS amplitude < 30 mV in the limb leads.

The ECG pattern of low QRS voltages (LQRSV) is represented by a QRS complex amplitude (measured from nadir to zenith) < 0.5 mV in limb leads (with an entire limb leads QRS amplitude < 30 mV). Low QRS voltages have been observed in several cardiac and non-cardiac pathological conditions associated with an increase in the electrical resistance within or outside the heart (e.g., infiltrative disease, pulmonary emphysema, obesity, or pericardial effusion) or a loss of cardiomyocytes with replacement fibrosis (e.g., ACM, cardiac sarcoidosis, healed myocarditis, or idiopathic non-ischemic left ventricular scar) (Figure 5) [90,91,92,93,94,95,96]. Interest has been growing in detecting LQRSV as a potential early marker of cardiomyopathies, especially ACM [97].

An LQRSV pattern may be unexpected in an athlete because physiological cardiac adaptation is usually associated with an increased QRS voltage [22]. However, in a cohort of 516 Olympic athletes [98], LQRSV was present in 4% of cases; athletes with LQRSV did not differ from other athletes in LV cavity size and mass, sex, and sport participation. However, more frequently, they showed PVCs with patterns suggesting an origin from the left or right free wall.

In a large population of young Italian competitive athletes [99], the prevalence of an isolated LQRSV pattern on ECG was 1.1%, which was significantly higher among elite than non-elite athletes. This pattern was associated with increasing age, body surface area, and body mass index, but not with sex, type of sport, or echocardiographic LV mass. One-third of the athletes with isolated LQRSV had PVCs during exercise testing. Further, CMR examination was performed in 5 of 24 athletes with isolated LQRSV because of ‘uncommon’ ventricular arrhythmias at exercise testing, resulting in abnormality in two of these five cases [99]. Therefore, the finding of LQRSV requires further evaluation because it may underlie heart disease.

## 6. Conclusions

Adaptation to exercise leads to several physiological changes that must be accurately distinguished from pathological findings. This distinction is challenging, as both false positive and potentially fatal false-negative interpretations can occur. The ECG is the most-effective and widely accessible tool for screening an athlete’s heart. It helps identify when further assessment is needed, thereby minimizing the risk of SCD while also limiting expensive diagnostic workups and unnecessary disqualification from competitive sports.

## Figures and Tables

**Table 1:** Expected electrocardiographic changes in athletes.

	Resistance Training 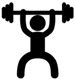	Mixed 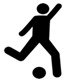	Endurance Training 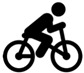
**HR**	Mild decrease (30–60 bpm)	Mild decrease (30–60 bpm)	Severe decrease (<30 bpm)
**RR interval**	Mild increase (<3 s)	Mild-to-moderate increase (≤3 s)	Severe increase (>3 s)
**P wave**	Generally normal <120 ms <2.5 mm in the limb leads <1.5 mm in the precordial leads	Generally normal <120 ms <2.5 mm in the limb leads <1.5 mm in the precordial leads	May be increased in amplitude >120 ms >2.5 mm in the limb leads >1.5 mm in the precordial leads
**PR interval**	No significant change (120–200 ms)	Moderate-to-huge increase (200–300 ms)	Severe increase (200–400 ms)
**QRS morphology**	No significant change	iRBBB	iRBBB-CRBBB
**QRS duration**	Generally normal (70–100 ms)	Generally normal (70–100 ms)	Mild increase (70–110 ms)
**QRS amplitude**	Mild-to-moderate increase	Moderate-to-huge increase	Severe increase (usually QRS voltage for RVH/LVH)
**QRS axis**	Generally normal (from −30 to −90°)	Mild left-axis deviation may be present (from −30 to −45°)	Left-axis deviation (from −45 to −90°)
**ST segment**	Generally normal	Early repolarization pattern can be seen, especially in black athletes	Early repolarization pattern can be seen, especially in black athletes
**T wave**	Generally normal	T-wave inversion possible in V1–V3, especially in adolescent athletes	T-wave inversion possible in V1–V3, especially in adolescent athletes

Abbreviations: CRBBB, complete right bundle branch block; HR, heart rate; iRBBB, incomplete right bundle branch block; LVH, left ventricular hypertrophy; RVH, right ventricular hypertrophy; V1–V3, precordial leads from V1 to V3.

**Table 2:** Areas of uncertainty in the interpretation of the athlete’s ECG.

Topic	Question
Low QRS voltages	Further studies are needed to assess the clinical significance of LQRSVs definitively and to evaluate whether and to what extent this ECG pattern may predict a heart muscle disease, regardless of other abnormal cardiac testing and clinical findings.
Premature ventricular contractions	Evaluation of PVCs is controversial, and excluding pathology may be challenging. The burden, the morphology, and the response to exercise should all be considered.
T-wave inversion	- It is difficult to provide a precise age cut-off to differentiate between the benign ‘juvenile pattern of repolarization’ and anterior repolarization abnormalities, which may represent the sign of underlying cardiomyopathy - The significance of T-wave inversion in the inferior leads is unknown but is probably a normal variant in black athletes, so clinicians should not misinterpret it as pathological findings
Early repolarization	Available data are too scarce to assume a causal relationship between the early repolarization model and the risk of malignant ventricular arrhythmias.
Complete right bundle branch block	CRBBB is generally considered a borderline finding by experts’ consensus as it may represent a more extreme right ventricular adaptation to exercise but also hide an underlying heart disease.

Abbreviations: CRBBB, complete right bundle branch block; LQRSVs: Low QRS voltages; PVCs: Premature ventricular contractions.
